# Editorial: Breeding approaches to improve woody plants’ resistance to pests and diseases

**DOI:** 10.3389/fpls.2026.1912861

**Published:** 2026-07-08

**Authors:** Rita Lourenço Costa, Elena Corredoira, Santiago Pereira Lorenzo

**Affiliations:** 1 National Institute for Agricultural and Veterinary Research (INIAV), Oeiras, Portugal; 2 Mision Biologica de Galicia Sede de Santiago de Compostela, Santiago de Compostela, Spain; 3 Universidade de Santiago de Compostela Escola Politecnica Superior de Enxenaria, Lugo, Spain

**Keywords:** breeding, genomics, genotyping, host-pathogen interactions, phenotyping

Tree diseases and pest invasions are among the greatest threats to forest ecosystems, fruit production, and the sustainability of perennial cropping systems worldwide. Climate change, increased global trade, and the emergence of new pathogens and pests are exacerbating these challenges, leading to substantial economic losses and ecological disruption. Developing tolerant woody plant varieties remains one of the most effective and environmentally sustainable strategies for mitigating these impacts. However, breeding woody species is inherently complex due to their long generation times, high levels of heterozygosity, and strong genotype-by-environment interactions. Recent advances in genomics, molecular breeding, transcriptomics, and biotechnology are nevertheless transforming the pace and precision with which resistance traits can be identified and deployed. This Research Topic was launched to provide a forum for studies addressing innovative breeding approaches and emerging technologies aimed at improving resistance to pests and diseases in forest trees, fruit crops, grapevines, and other woody species.

The ten articles included in this Research Topic illustrate the diversity of approaches currently being used to accelerate resistance breeding and improve our understanding of host–pathogen interactions in woody plants. Together, they highlight the integration of traditional breeding, germplasm evaluation, quantitative genetics, molecular markers, and functional genomics to address major phytosanitary challenges.

Several contributions focus on grapevine, a crop that faces numerous disease threats worldwide. In *Screening of Galician grapevine varieties by SNPs, phenotypic traits, and phytopathology*, Díaz-Fernández et al. combined molecular and phenotypic characterization to evaluate regional germplasm, demonstrating the value of local genetic resources for breeding and conservation. Complementing this work, Ricciardi et al., in *Novel loci associated with resistance to downy and powdery mildew in grapevine*, identified new genomic regions associated with resistance to two of the most economically important grapevine diseases. Their findings contribute valuable markers that can facilitate marker-assisted breeding and accelerate the development of resistant cultivars.

Chestnut improvement is represented by two studies addressing one of the most devastating diseases affecting the genus. In *Mapping QTLs for blight resistance and morpho-phenological traits in inter-species hybrid families of chestnut* (*Castanea* spp.), Fan et al. identified genomic regions associated with resistance and adaptive traits in hybrid populations, providing useful tools for breeding programs. At the molecular level, Fernandes et al., in *Dual transcriptomic analysis reveals early induced Castanea defense-related genes and Phytophthora cinnamomi effectors*, examined the early interaction between chestnut and the causal agent of ink disease. Their work revealed candidate defense genes and pathogen effectors that improve our understanding of tolerance mechanisms and may support future breeding and biotechnology strategies.

The Research Topic also includes important contributions addressing resistance breeding in *Coryllus avelana* Jacobs et al., in *Variable response of eastern filbert blight resistance sources in New Jersey*, demonstrated that resistance expression can vary among genetic backgrounds, emphasizing the importance of validating resistance sources across breeding populations. In a second study, *Eastern filbert blight resistant Corylus avellana identified from 20 years of germplasm introduction and evaluation at Rutgers University, New Jersey, USA*, the same research group reported the identification of valuable resistant germplasm after two decades of systematic evaluation. These findings illustrate the enduring importance of long-term breeding efforts and germplasm conservation.

Forest tree species are represented through both applied breeding and broader strategic perspectives. Cordeiro et al., in *Breeding Alnus species for resistance to Phytophthora disease in the Iberian Peninsula*, reviewed current knowledge on alder resistance breeding and discussed opportunities and challenges associated with managing *Phytophthora* diseases in riparian ecosystems. The review highlights the importance of integrating conservation and breeding objectives to protect vulnerable native populations.

The contribution by Fumia et al., *Acacia koa seedling disease tolerance and vigor driven by breeding orchard size*, examined how breeding population structure influences disease tolerance and seedling performance. Their results suggest that breeding orchard design can have important consequences for maintaining genetic diversity while improving resistance-related traits.

The global importance of genotype-by-environment interactions is addressed in *Global Coffea arabica variety trials reveal genotype-by-environment interactions in resistance to coffee leaf rust (Hemileia vastatrix)* by (Berny Mier y Teran et al.) Through multi-environment trials across coffee-growing regions, the authors demonstrated that resistance performance depends strongly on environmental context, reinforcing the need for international testing networks and location-specific breeding strategies.

Beyond direct resistance breeding, Xu et al., in *Another perspective of strain selection based on functional traits: construction and evaluation of a key complex index for endangered species plantation*, proposed an integrated framework for evaluating planting material using functional traits. Although developed in the context of endangered species plantations, their approach provides insights into how multiple adaptive characteristics can be combined to support resilient woody plant populations.

Collectively, the studies in this Research Topic demonstrate that improving of tolerance/resistance in woody plants requires a multidisciplinary approach that spans germplasm evaluation, quantitative genetics, molecular marker development, transcriptomics, and long-term field testing. The contributions also emphasize that resistance is rarely a simple trait; rather, it emerges from complex interactions among host genetics, pathogen biology, and environmental conditions. Advances in genomics, high-throughput phenotyping, and bioinformatics are creating unprecedented opportunities to dissect these interactions and accelerate breeding progress.

As global pressures from emerging pathogens, invasive pests, and climate change continue to intensify, the development of resistant woody plant varieties will remain a critical priority for sustainable agriculture and forestry. We hope that the studies presented in this Research Topic will stimulate further research, foster international collaboration, and contribute to the deployment of resilient woody crops and forest trees capable of meeting future challenges.

